# Pre-Historic Dental Diseases

**Published:** 1884-01

**Authors:** Wm. H. Trueman

**Affiliations:** No. 900 Spruce Street, Philadelphia


No. 900 Spruce Street,

Philadelphia, December 15th, 1883.

*Editor Independent Practitioner:*

I have paid two visits to the Museum of the Academy of Natural Sciences in this city, for the purpose of examining some of the skulls there, to see if they bore evidence of dental disease. Many of them are in a condition to make them of little value from a dental standpoint, the teeth having been lost, and the alveolar process so broken that nothing can be learned from them. My examination of the rest was necessarily but cursory, but I hope at some day to make it more thorough, and then the INDEPENDENT PRACTITIONER shall have the results.

A day or two since I gave the case containing the Peruvian skulls especial attention, and a slight examination revealed extensive loss of teeth, irregularity, evidences of alveolar abscess, caries in various locations, and deposits of salivary calculus, such as we see at the present day. Some dentures were apparently good; in others there was a loss of, or changed condition of the process, that may have been the result of disease, or may have been caused after death by the soil in which they were buried, or climatic influence, or by time. I rather think it posthumous. I was surprised to find but few North American Indians with perfect dentures, nearly all as they lay on the shelves exhibiting evidence of some form of dental defect. In conclusion, the examination, although quite cursory, was conclusive in one respect, and that is in showing the importance of giving the subject far more attention than it has yet received. Not only should the skulls be carefully examined, but it is equally important that the history of the races to which they belong should be carefully studied, their origin, habits, diet, manner of life, etc., noted and compared, so as to see what influence these have had upon the dentures they have left behind them.

Very respectfully,

WM. H. TRUEMAN, D. D. S.

